# Relation between body mass index and depression: a structural equation modeling approach

**DOI:** 10.1186/1471-2288-7-17

**Published:** 2007-04-30

**Authors:** Alina Dragan, Noori Akhtar-Danesh

**Affiliations:** 1 Nursing Health Services Research Unit, McMaster University, Canada; 2 School of Nursing and Department of Clinical Epidemiology & Biostatistics, McMaster University, Canada

## Abstract

**Background:**

Obesity and depression are two major diseases which are associated with many other health problems such as hypertension, dyslipidemia, diabetes mellitus, coronary heart disease, stroke, myocardial infarction, heart failure in patients with systolic hypertension, low bone mineral density and increased mortality. Both diseases share common health complications but there are inconsistent findings concerning the relationship between obesity and depression. In this work we used the *structural equation modeling *(SEM) technique to examine the relation between body mass index (BMI), as a proxy for obesity, and depression using the Canadian Community Health Survey, Cycle 1.2.

**Methods:**

In this SEM model we postulate that 1) BMI and depression are directly related, 2) BMI is directly affected by the physical activity and, 3)depression is directly influenced by stress. SEM was also used to assess the relation between BMI and depression separately for males and females.

**Results:**

The results indicate that higher BMI is associated with more severe form of depression. On the other hand, the more severe form of depression may result in less weight gain. However, the association between depression and BMI is gender dependent. In males, the higher BMI may result in a more severe form of depression while in females the relation may not be the same. Also, there was a negative relationship between physical activity and BMI.

**Conclusion:**

In general, use of SEM method showed that the two major diseases, obesity and depression, are associated but the form of the relation is different among males and females. More research is necessary to further understand the complexity of the relationship between obesity and depression. It also demonstrated that SEM is a feasible technique for modeling the relation between obesity and depression.

## Background

Obesity and depression are two major diseases associated with numerous health complications [1]. Obesity is linked with hypertension, dyslipidemia, diabetes mellitus, coronary heart disease, stroke, as well as increased all cause mortality [2]. Depression contributes to increased risk of coronary heart disease, myocardial infarction, heart failure in patients with systolic hypertension, low bone mineral density, and increased mortality [3-8].

Both diseases share common health complications but there is inconsistent findings concerning the relationship between obesity and depression. Some studies concluded that there was no relation between obesity and depression [9,10], while others reported that obese people had higher risk of depression [11,12] or that heavier people were less depressed [13,14]. Goodman and Whitaker [15] showed that depressed adolescents are at increased risk for the development and persistence of obesity later in their life and Pine et al. [16] concluded that depression in childhood was positively associated with BMI during adulthood. In spite of the inconsistent findings overall it is believed that psychological distresses caused by obesity may lead to depression [17]. Other researchers, for instance Rosmond [18], have even suggested that obesity and depression might be the same disease with different manifestations. The relation between obesity and depression could depend on factors such as sex, level of obesity, level of depression, socio-economic status and family history of depression [17].

In this article we used the *structural equation modeling *(SEM) technique to assess the relation between BMI (as a proxy for obesity) and depression in a sample of 12,376 individuals from the province of Ontario as a subset of the 2002 Canadian Community Health Survey, Cycle 1.2 (CCHS-1.2) dataset. The main objective was to examine the potential of the SEM technique for such complex analysis. This sample size is large enough to disentangle the complexity of the relation between obesity and depression. In addition, the results could be used as an approximation for Canadian population given that Ontario represents about 40% of the total Canadian population [19].

## Methods

### Data source

The CCHS-1.2 is a cross-sectional survey that contains information related to health status, health care utilization and health determinants for the Canadian population. The survey is based on a complex design, with stratification and multiple stages of selection, and unequal probabilities of selection to ensure adequate representation of young persons (15 to 24 years) and seniors (65 years and over) [20]. The questionnaire was administered on the sample units selected from the area frames using the computer-assisted interviewing (CAI) method. One person aged 15 years and over was randomly selected from the sampled households. The dataset contains information from 36,984 individuals with a response rate of 77.0% in Canada of which 12,376 respondents were from Ontario. There were 76 pregnant women who were excluded from the analysis.

### Variables

The following variables have been suggested in the literature to be associated with the relation between depression and obesity:

#### Body mass index (BMI)

The CCHS-1.2 survey used the respondents' self-reported measurements of height and weight to calculate the body mass index (BMI) which is an index of weight-to-squared height (*kg */*m*^2^). In this analysis BMI has been used as a continuous variable as reported in the dataset.

#### Depression

*Persistence of Major Depressive Episode *is a continuous variable that identifies the longest period associated with a major depressive episode experienced by the respondent. We used this variable as the main proxy for depression.

#### Physical activity

To derive a physical activity index, the energy expenditure (EE) of participants in their leisure activities was estimated using the frequency and time per physical activity session as well as the metabolic energy cost (MET) value. This value is expressed as a multiple of the resting metabolic rate [21,22]. In this survey respondents were asked to specify the intensity level of their activities; therefore the MET values adopted correspond to the low intensity value of each activity.

#### Stress management

This variable was approximated by *ability to handle unexpected and difficult problems *in the dataset. Participants were asked to rate their ability in handling unexpected and difficult problems such as a family or personal crisis on a 5-point Likert scale from excellent to poor so that higher scores relate to decreased ability in handling unexpected and difficult problems.

#### Socioeconomic status (SES)

Socioeconomic status is frequently measured by job status, income, and education [23,24]. We used a factor analysis technique to extract a latent variable from the variables of *job status during the past year*, *multiple job status*, *income per household*, *income per person*, and *education*. As a result, *education *did not load significantly on the factor; therefore, we removed it and made the SES factor with the other four variables.

#### Eating habits – eating attitudes test (EAT) index

This variable measures the extent of the symptoms and concerns characteristic of eating disorders [25]. Higher scores of EAT index indicate higher risk of eating disorder.

#### Relatives with depression

This variable is a measure of the number of close relatives, including biological parents, siblings, and children who ever had at least one episode of being depressed.

### Statistical analysis

The *structural equation modeling *is a method for representing, estimating and testing a theoretical network of mostly linear relations between variables that may be either directly observable or unobservable and may only be measured imperfectly. It is a generalization of both regression and factor analysis and comprises most of the linear modeling methods as "special cases". The procedure places emphasis on covariance structures rather than cases. The fundamental hypothesis in using SEM is that the covariance matrix of the observed variables is a function of a set of parameters. If the model is correct and the parameters are known, then the population covariance matrix would be exactly reproduced by SEM (except for sampling variation). SEM proceeds by assessing whether a sample covariance or correlation matrix is consistent with a hypothetical matrix implied by the model. The inputs are either raw data or sample moments computed from the data, and a model to be evaluated. The sample moments will include either correlations or variances and covariances. It may also include means and higher order moments [26].

The general SEM model can be decomposed into two submodels: a measurement model and a structural model. The measurement model defines relations between the observed and unobserved latent variables. The structural model defines relations among the unobserved variables by specifying the pattern by which particular latent variables directly or indirectly influence some other latent variables in the model.

SEM is mainly a confirmatory technique rather than exploratory and is more likely to be used to determine whether a certain model is valid, rather than to find a suitable model. However, SEM analysis often involves a certain degree of exploratory analysis. By convention, when graphically representing the model the observed variables are enclosed by rectangles or squares and latent variables are enclosed by ovals or circles. Residuals are always unobserved and are represented by ovals or circles.

In this work to evaluate the goodness-of-fit of a model the *root mean square error of approximation *(RMSEA) statistic and the *comparative fit index *(CFI) were used as these are the most commonly used indices [26]. The RMSEA estimates the lack of fit in a model compared to a saturated model. The estimated RMSEA is given by:

estimated RMSEA=F^0dfmodel
 MathType@MTEF@5@5@+=feaafiart1ev1aaatCvAUfKttLearuWrP9MDH5MBPbIqV92AaeXatLxBI9gBaebbnrfifHhDYfgasaacH8akY=wiFfYdH8Gipec8Eeeu0xXdbba9frFj0=OqFfea0dXdd9vqai=hGuQ8kuc9pgc9s8qqaq=dirpe0xb9q8qiLsFr0=vr0=vr0dc8meaabaqaciaacaGaaeqabaqabeGadaaakeaacqqGLbqzcqqGZbWCcqqG0baDcqqGPbqAcqqGTbqBcqqGHbqycqqG0baDcqqGLbqzcqqGKbazcqqGGaaicqqGsbGucqqGnbqtcqqGtbWucqqGfbqrcqqGbbqqcqGH9aqpdaGcaaqaamaalaaabaGafmOrayKbaKaadaWgaaWcbaGaeGimaadabeaaaOqaaiabdsgaKjabdAgaMnaaBaaaleaacqqGTbqBcqqGVbWBcqqGKbazcqqGLbqzcqqGSbaBaeqaaaaaaeqaaaaa@4C4B@

where F^0=Max(χmodel2−dfmodelN,0)
 MathType@MTEF@5@5@+=feaafiart1ev1aaatCvAUfKttLearuWrP9MDH5MBPbIqV92AaeXatLxBI9gBaebbnrfifHhDYfgasaacH8akY=wiFfYdH8Gipec8Eeeu0xXdbba9frFj0=OqFfea0dXdd9vqai=hGuQ8kuc9pgc9s8qqaq=dirpe0xb9q8qiLsFr0=vr0=vr0dc8meaabaqaciaacaGaaeqabaqabeGadaaakeaacuWGgbGrgaqcamaaBaaaleaacqaIWaamaeqaaOGaeyypa0Jaemyta0KaemyyaeMaemiEaGNaeiikaGYaaSaaaeaaiiGacqWFhpWydaqhaaWcbaGaeeyBa0Maee4Ba8MaeeizaqMaeeyzauMaeeiBaWgabaGaeGOmaidaaOGaeyOeI0IaemizaqMaemOzay2aaSbaaSqaaiabb2gaTjabb+gaVjabbsgaKjabbwgaLjabbYgaSbqabaaakeaacqWGobGtaaGaeiilaWIaeGimaaJaeiykaKcaaa@4CD1@ and *N *is the sample size [27]. For a perfect model F^0
 MathType@MTEF@5@5@+=feaafiart1ev1aaatCvAUfKttLearuWrP9MDH5MBPbIqV92AaeXatLxBI9gBaebbnrfifHhDYfgasaacH8akY=wiFfYdH8Gipec8Eeeu0xXdbba9frFj0=OqFfea0dXdd9vqai=hGuQ8kuc9pgc9s8qqaq=dirpe0xb9q8qiLsFr0=vr0=vr0dc8meaabaqaciaacaGaaeqabaqabeGadaaakeaacuWGgbGrgaqcamaaBaaaleaacqaIWaamaeqaaaaa@2EEB@ = 0. Values of RMSEA of 0.06 or less indicate a good-fitting model and a value larger than 0.10 is indicative of a poor model.

The *comparative fit index *(CFI) assesses fit relative to other models. It employs the noncentral *χ*^2 ^distribution with noncentrality parameters, *τ*_*i*_. The larger *τ*_*i *_indicates the greater model misspecification. For a perfect model *τ*_*i *_= 0. The CFI is defined as:

CFI=1−τmodelτindep
 MathType@MTEF@5@5@+=feaafiart1ev1aaatCvAUfKttLearuWrP9MDH5MBPbIqV92AaeXatLxBI9gBaebbnrfifHhDYfgasaacH8akY=wiFfYdH8Gipec8Eeeu0xXdbba9frFj0=OqFfea0dXdd9vqai=hGuQ8kuc9pgc9s8qqaq=dirpe0xb9q8qiLsFr0=vr0=vr0dc8meaabaqaciaacaGaaeqabaqabeGadaaakeaacqqGdbWqcqqGgbGrcqqGjbqscqGH9aqpcqaIXaqmcqGHsisldaWcaaqaaGGaciab=r8a0naaBaaaleaacqqGTbqBcqqGVbWBcqqGKbazcqqGLbqzcqqGSbaBaeqaaaGcbaGae8hXdq3aaSbaaSqaaiabbMgaPjabb6gaUjabbsgaKjabbwgaLjabbchaWbqabaaaaaaa@444E@

with

*τ*_indep _= χindep2
 MathType@MTEF@5@5@+=feaafiart1ev1aaatCvAUfKttLearuWrP9MDH5MBPbIqV92AaeXatLxBI9gBaebbnrfifHhDYfgasaacH8akY=wiFfYdH8Gipec8Eeeu0xXdbba9frFj0=OqFfea0dXdd9vqai=hGuQ8kuc9pgc9s8qqaq=dirpe0xb9q8qiLsFr0=vr0=vr0dc8meaabaqaciaacaGaaeqabaqabeGadaaakeaaiiGacqWFhpWydaqhaaWcbaGaeeyAaKMaeeOBa4MaeeizaqMaeeyzauMaeeiCaahabaGaeGOmaidaaaaa@364C@ - *df*_indep _and *τ*_model _= χmodel2
 MathType@MTEF@5@5@+=feaafiart1ev1aaatCvAUfKttLearuWrP9MDH5MBPbIqV92AaeXatLxBI9gBaebbnrfifHhDYfgasaacH8akY=wiFfYdH8Gipec8Eeeu0xXdbba9frFj0=OqFfea0dXdd9vqai=hGuQ8kuc9pgc9s8qqaq=dirpe0xb9q8qiLsFr0=vr0=vr0dc8meaabaqaciaacaGaaeqabaqabeGadaaakeaaiiGacqWFhpWydaqhaaWcbaGaeeyBa0Maee4Ba8MaeeizaqMaeeyzauMaeeiBaWgabaGaeGOmaidaaaaa@364E@ - *df*_model_.

The parameter *τ*_indep _= χindep2
 MathType@MTEF@5@5@+=feaafiart1ev1aaatCvAUfKttLearuWrP9MDH5MBPbIqV92AaeXatLxBI9gBaebbnrfifHhDYfgasaacH8akY=wiFfYdH8Gipec8Eeeu0xXdbba9frFj0=OqFfea0dXdd9vqai=hGuQ8kuc9pgc9s8qqaq=dirpe0xb9q8qiLsFr0=vr0=vr0dc8meaabaqaciaacaGaaeqabaqabeGadaaakeaaiiGacqWFhpWydaqhaaWcbaGaeeyAaKMaeeOBa4MaeeizaqMaeeyzauMaeeiCaahabaGaeGOmaidaaaaa@364C@ - *df*_indep _refers to the *independence model *which assumes zero population covariances among the observed variables [28]. CFI values greater than 0.90 indicate reasonably good fit of the model [29]. The analysis were performed using the AMOS software [30].

### The Stunkard model

Stunkard et al. [31] suggested a model which encompasses a "genetic correlation" and an "environmental correlation" between depression and obesity. The "genetic correlation" refers to the likelihood that there might be a set of genes that promote both depression and obesity, while the "environmental correlation" underlines the possibility of existence of "common life experiences" that may promote both diseases. Although this model does not completely explain the physiological pathways between the two diseases, it has been used as an empirical framework in studies of genetic epidemiology [32].

**Figure 1:**
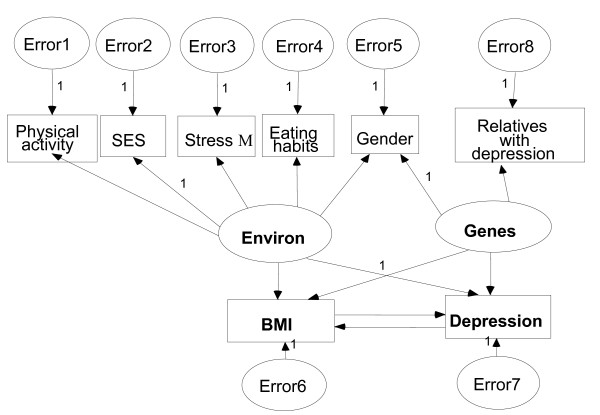
Model 1 – SEM model with two latent variables of Environ and Genes (Stress M = Stress Management).

We started the analysis with an adaptation of Stunkard's model. This model consists of two unobserved latent variables of *Environ *(for environment) and *Genes *and eight observed variables (Figure 1). The observed variables load on the latent variables (factors) in the following pattern:

a) *Physical activity, SES, stress management, eating habits *and *gender *which are usually considered as the components of the environment load on the first factor, *Environ*.

b) *Gender *and *relatives with depression *load on the second factor, *Genes*.

c) Both *BMI *and *depression *regress on the aforementioned factors.

Measurement errors associated with each observed variable were added with the assumption that these error terms were independent from each other.

This hypothesized model is a *non-recursive model*, that is, it is a model with two structural equations where the dependent variable of each equation appears as a predictor variable in the other equation. In this model *BMI *and *depression *form a feedback loop; meaning that we can follow the path between these two variables on an infinite number of times without having to return to the other variables. This model states that *BMI *is directly influenced by *depression *and vice versa.

## Results

A summary of descriptive statistics is given in Table 1. As this table shows about 50 percent of Ontarian suffer from overweight or obesity and approximately 12 percent of the respondents reported experiencing some period of major depressive episode. In this dataset variables of *eating habit *and *relatives with depression *have large numbers of missing values.

**Table 1:** Descriptive statistics of variables used in the analysis

**Variables**	**N (%)**	**Mean**	**Std. Deviation**	**Min**	**Max**
**Gender**	**12376**	-	-	-	-
Males	5660 (45.3)	-	-	-	-
Females	6716 (54.3)	-	-	-	-
**Body Mass Index**	**10961**^§^	25.75	4.84	9.60	57.80
Under/Normal weight	5449 (49.7)	-	-	-	-
Overweight	3710 (33.8)	-	-	-	-
Obese	1802 (16.4)	-	-	-	-
**Depression (# years with MDE-lifetime)**	**12282**^§^	0.41	8.20	0	67.00
No depression (0 years)	10826 (88.1)	-	-	-	-
Yes depression	1456 (11.9)	-	-	-	-
**Physical Activity**	**12375**^§^	2.27	2.34	0	28.70
**Stress Management**	**12352**^§^	2.31	0.93	1	5
**Eating Habits**	**1812**^§^	10.63	8.85	0	62
**Relatives with Depression**	**1512**^§^	1.76	3.02	0	55

§ the number is different from the total of 12376 because of missing values

Of great importance in SEM is the extent to which the hypothesized model fits the dataset. Using SEM technique, Model 1 (see Figure 1) had values less than the minimum acceptable level and the goodness-of-fit indices proved not to be a good fit to the dataset. In particular, it showed that *Genes *is not a good construct of *gender *and *relatives with depression*.

In an effort to improve the model the variable *relatives with depression *was eliminated due to large numbers of missing data and the connection between *gender *and *depression *was dropped due to its non-significant path (Model 2). However, these changes did not improve the model satisfactorily. Consequently the resulting third model had one latent variable, *Environ*, with *gender *connected to it directly. The model contained 6 observed variables: *physical activity, SES, stress management, gender, BMI *and *depression *and one latent variable, *Environ*. In this third model it was hypothesized that *BMI *is directly affected by *physical activity *and *depression *is directly influenced by *stress*, as previously reported in the literature [33].

For this model the estimated RMSEA is 0.047 with the 90% confidence interval of (0.040, 0.054) and the p-value for the test of closeness of fit of 0.776. The interpretation of the confidence interval indicates that with 90% confidence the true RMSEA value in the population falls within the bounds of 0.040 and 0.054, and therefore represents a good degree of precision. Given that the upper bound of the 90% confidence interval is less than the suggested value of 0.06 [34], and the probability value associated with this test of close fit is > 0.50 (i.e. 0.776), it can be concluded that the hypothesized model fits the data well. In addition, the CFI value is 0.898 which indicates an acceptable level for model fitting. Also, the factor loadings and path coefficients for this model are significantly different from zero and there is no near-zero standard errors for the factor loadings and path coefficients (Table 2). Of great interest in the analysis are the path coefficients that constitute the structural portion of the model. The path coefficients for the path from *BMI *to *depression*, from *depressio*n to *BMI*, from *Environ *to *BMI *and *Environ *to *depression *are all significant. In addition, AMOS estimates a *stability index *for each model. An index value between -1 and 1 indicates a stable model [26]. For Model 3 a stability index of 0.042 indicates that the system is indeed stable.

**Table 2:** Regression coefficients for Model 3

	**Estimate**	**SE**	CR*	**P**
Phys.Activ ← Environ	1.053	0.101	10.387	< 0.001
Stress M ← Environ	-0.548	0.044	-12.420	< 0.001
BMI ← Environ	1.738	0.256	6.781	< 0.001
Depression ← Environ	-0.963	0.141	-6.821	< 0.001
BMI ← Phys.Activ	-0.252	0.021	-11.859	< 0.001
Depression ← Stress M	0.274	0.034	8.091	< 0.001
SES ← Environ	1.000			
Gender ← Environ	-0.600	0.048	-12.385	< 0.001
BMI ← Depression	-0.282	0.116	-2.439	0.015
Depression ← BMI	0.150	0.045	3.359	< 0.001

* Critical ratio (the estimate divided by its standard error)

### Subgroup analysis

**Figure 2:**
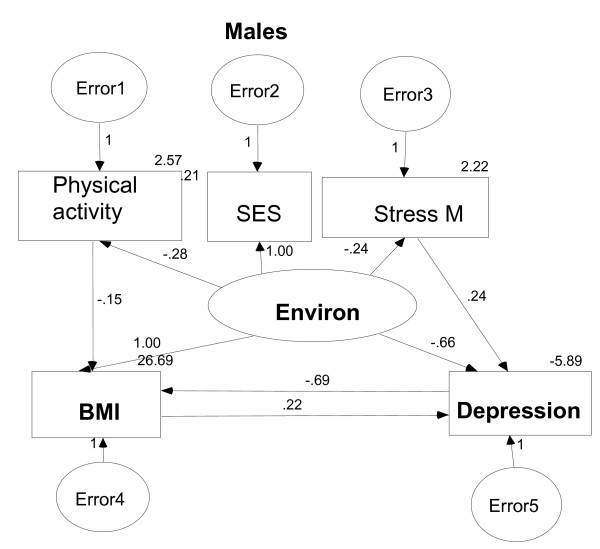
SEM model for male participants (Stress M = Stress Management).

Model 3 indicates that there is a relationship between BMI and depression and the effect of gender on this relationship is accounted for through the latent variable of *Environ *(see Table 2). This result indicates that relation between depression and obesity may be gender dependent. Therefore, we used SEM to assess the relation between BMI and depression separately for males and females. From the total sample of 12,376 respondents there were 5,660 males and 6,716 females. In subgroup analysis, Model 3 fit very well to the male group (Figure 2) with the goodness of fit indices of RMSEA = 0.056 and CFI = 0.890. However, the same model was not appropriate for the female participants.

**Figure 3:**
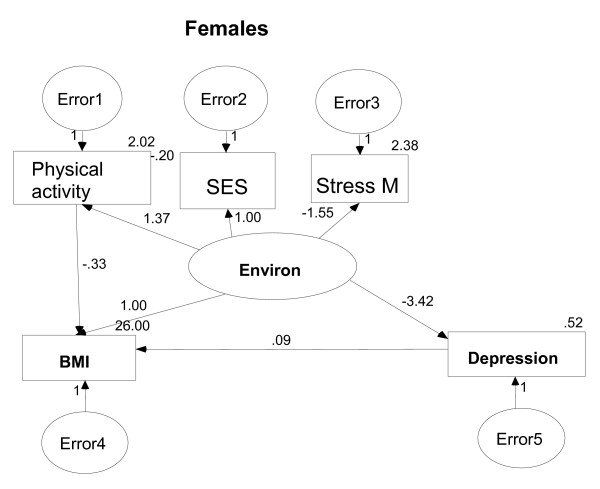
SEM model for female participants (Stress M = Stress Management).

In searching for a suitable model for females we dropped the connection between *stress management *and *depression *and the arrow from *BMI *to *depression *because they were not statistically significant. In this final model (Figure 3) all estimates were indeed significant and the goodness-of- fit indices were at acceptable level; RMSEA = 0.038 with 90% CI= (0.028, 0.048) and CFI = 0.879.

## Discussion

This work is based on a cross-sectional dataset and we had no information about history of obesity and depression in the family, history of debilitating diseases in the family and household, and adverse childhood experiences. Therefore, any statistically significant relation between variables cannot necessarily indicate causality. In addition, while SEM may produce a well fitting model, it may not be unique and there can be other reasonable models for the same dataset. Limitations with the AMOS software did not allow for the use of appropriate sampling weights for estimations and modeling. Nevertheless, this modeling has generated some useful conclusions which may be used to inform future studies.

Although BMI is not a direct measure of body fat or lean tissue, it is the most widely investigated indicator of health problems associated with under-weight and overweight statuses. However, BMI as an indicator of risk may have limitations for individuals or populations who are very tall or very short or who have very long or short limb lengths in relation to trunk measurement [35].

Results of this analysis indicate that BMI and depression are associated and that this relation is gender dependent which is supported by some other studies [36,37]. In general, it can be concluded that higher BMI may result in more severe forms of depression (see Table 2) which is consistent with some other recent findings [11,12]. On the other hand, the same conclusion may not be drawn for the effect of depression on BMI, therefore, a more severe form of depression may not result in more weight gain. The subgroup analysis based on gender showed that in males the higher BMI is related to the more severe form of depression, and the higher is the level of depression the lower is the BMI. These seemingly contradictory results about the relation between BMI and depression are in accordance with studies conducted by Palinkas et al. [13] and Stewart and Brook [14] and show the ability of the SEM method to differentiate directions of effects in a complicated modeling procedure. A possible explanation might be that new medications used to treat patients with depression have reduced/no weight gain side effect when compared to previous treatment options. However this is only a hypothesis as information regarding the treatment of these respondents was not available.

Another important result is the negative relationship between physical activity and BMI, which translates into "more physical activity – less weight problems". Model 3 also indicated that stress management had a direct relation with depression. The high score on the *stress management *means less ability to handle stress, indicating that individuals with reduced ability to handle stress suffer more from depression. The same relation was seen when males were analysed separately from females.

Among females stress management did not indicate a direct relation with depression and the path from *obesity *to *depression *was not statistically significant. In addition, the analysis did not show any relation between *eating habits *and *relatives with depression *and the outcome variables of *BMI *and *depression*. This lack of relationship may be explained by the large numbers of missing values in the variables of *eating habits *and *relatives with depression*.

In conclusion, this work shows that SEM can be used as an appropriate method to disentangle the complexity of the relation between obesity and depression. Further research is needed to better understand the structure of such complexity. Interestingly, this work showed that obesity and depression are associated but the form of the relation is different among males and females.

## Competing interests

The author(s) declare that they have no competing interests.

## Authors' contributions

Both authors contributed equally in designing the study and drafting the manuscript. The analysis was carried out by AD.

## Pre-publication history

The pre-publication history for this paper can be accessed here:


